# Supplementary material for: A social niche breadth score reveals niche range strategies of generalists and specialists
Source: Nat Ecol Evol. 2023 Apr 3;7(5):768–81. doi: 10.1038/s41559-023-02027-7 (PMC10172124; doi:10.1038/s41559-023-02027-7)
Supplement: Supplementary file 1 — Supplementary results and discussion, Figs. 1–19 and references. [file 41559_2023_2027_MOESM1_ESM.pdf]

nature ecology & evolution

Article https://doi.org/10.1038/s41559-023-02027-7

A social niche breadth score reveals niche range strategies of generalists and specialists

In the format provided by the authors and unedited

Supplementary Information

Supplementary Results and discussion

A cross-biome dataset

We compiled a diverse set of 22,518 environmental sequencing samples from 592 studies, spanning 140 annotated biomes across a wide geographical range based on the MGnify resource[1] (**Supp. Fig. 1**, **Fig. 1, Suppl. Data File 1**, see **Methods** for selection criteria). MGnify uses standardised pipelines to process environmental sequencing datasets, allowing for the comparison of samples across a wide range of different environments, studies, and experiment types. We only included taxonomic profiles that were constructed with the 4.1 pipeline version of MGnify and based on the small subunit (SSU) rRNA gene. Because these taxonomic classifications are all based on queries with the same rRNA gene models[1] that can be found in both targeted amplicon and shotgun studies, we included sequencing samples from amplicon, metagenomic, metatranscriptomic, and even the elusive 'unknown' experiment types.

Samples from similar annotated biomes clustered together based on microbial composition, despite the samples coming from vastly different locations and study designs including experiment type (**Fig. 1d**, **Supp. Fig. 2a**, **Supp. Fig. 3**). Samples were mostly separated by association to a vertebrate host versus free-living habitats, and saline versus non-saline habitats[2–5] (**Supp. Fig. 4a**, **Supp. Fig. 2b**). An exception are fish, whose foregut and intestinal microbiomes were more similar to microbiomes from aquatic habitats than to those in other vertebrate guts (**Supp. Fig. 3**). Within free-living habitats, saline samples differed from non-saline samples including soils. Aquatic sediments resembled their saline or non-saline provenance. In line with earlier findings[3], invertebrate-associated samples clustered together with free-living samples and not with vertebrate-associated samples. Invertebrates in our dataset include sponges, molluscs, *Cnidaria*, and *Echinodermata* whose internal microbiomes are in direct or semi-direct contact with the surrounding environment, and marine arthropods like *Calanus finmarchicus* (**Supp. Data File 1**).

Host-associated samples typically had lower taxa richness and alpha diversity than free-living samples (**Supp. Fig. 4b,c**, **Supp. Fig. 2c-e**, **Supp. Fig. 5a**). Rhizospheres have been shown to resemble soils in terms of richness[4] and we also observed this (**Fig. 1e**). Notably, annotated biomes with a high mean alpha diversity had a low beta diversity, while annotated biomes with low mean alpha diversity had either low or high beta diversity (**Fig. 1f**).

While taxa richness increases towards low taxonomic ranks, richness in the microbiomes was lower at the species rank and in some cases also at the genus rank than at higher ranks (**Fig. 1e**). This anomaly reflects the still low classification rate of the organisms in natural environments at the species and genus ranks. In addition, low rank classifications more easily fall below our detection limit of 1 / 10,000 than a higher rank classification whose abundance is the sum of its lower ranks.

Quantifying microbial social niche breadth

To quantify the range of habitats in which a microbial taxon is found we formulated a social niche breadth score that is data-driven and independent of human-defined biome annotations. To do this, we calculated the dissimilarity between taxonomic profiles of pairs of samples (see below), and defined the social niche breadth" (SNB) of a taxon as the mean pairwise dissimilarity between the microbial communities of the samples where it is found. Thus, taxa that always occur in samples with similar microbial composition have a low SNB ("social specialists"), whereas taxa that occur in dissimilar samples have a high SNB ("social generalists").

Benchmarking microbiome dissimilarity measures

To arrive at a quantitative niche breadth definition that optimally reflects annotated biomes as recognised by the research community, we benchmarked 150 different microbiome dissimilarity measures for their correspondence with the biome annotations of the underlying datasets. Many dissimilarity measures have been proposed in ecological literature, each with their own merit. For example, the Aitchison distance[6] is relevant for microbiomes because it takes the inherent compositionally of sequencing data into account[7], while the Unifrac distance considers phylogenetic (or taxonomic) information[8]. Measures that take relative abundances into account (weighted measures) better reflect quantitative relationships between taxa than unweighted measures, but put less emphasis on low abundant taxa that might be instrumental for ecosystem functioning[9]. Other factors of importance include the taxonomic rank of comparison, as well as the method for handling unknowns (sequences that cannot be classified).

We calculated ten ecological dissimilarity measures between all 253,518,903 sample pairs at six taxonomic ranks and with four different methods for dealing with unknowns (see **Methods**), totalling 150 different measures (**Supp. Fig. 6a-d**). The dissimilarity measures are based on: Aitchison distance, Bray-Curtis dissimilarity, Sørensen-Dice coefficient, Jaccard distance, weighted Jaccard distance, Kendalls τ_b coefficient, Pearson correlation coefficient, Spearman's rank correlation coefficient, unweighted Unifrac distance, and weighted Unifrac distance. Most measures are true distance or dissimilarity measures whereas the correlation and Sørensen-Dice coefficients were converted to a scale from 0 to 1, with 0 being compositionally similar (see **Methods**).

Since the MGnify taxonomic annotation pipeline depends on sequence similarity to a reference database and environmental sequencing studies contain both known and unknown taxa[10], many reads in a sample are not classified at all ranks (superkingdom, phylum, class, order, family, genus, species). Furthermore,

taxonomy is incomplete, with lower rank classifications sometimes present but intermediate ones missing. For example, taxonomy of the phylum *Cyanobacteria* is debated[11] and currently only one order has a taxonomic annotation at rank class in NCBI taxonomy. Likewise, the genus *Methyloceanibacter* of the order *Rhizobiales* does not have a family annotation yet[12]. For these reasons, we calculated the ecological dissimilarity measures with four different methods for dealing with unknowns (see **Methods**). Approach (i) substitutes unknowns at the considered rank with known lower or higher rank classifications. Whereas this approach has the benefit that all reads are considered, it potentially clusters different unknown taxa under the same higher rank umbrella, or may divide a would-be single taxon into multiple lower rank groups. Approach (ii) only compares reads that are annotated a the considered rank. This allows for a robust taxonomic comparison when samples contain unknowns, at the expense of using all reads. Approach (iii) is similar to approach (i) but removes unknowns that do not have lower rank classifications if they are shared between the two samples. The rationale is that for these taxa it is unknown if they are the same or different for the rank of interest. Finally, for the Unifrac distances we allow for placement of unknown taxa at different ranks in the tree (approach (iv)), with lower rank classifications artificially placed at the considered rank, and higher rank classifications kept. This allows for all reads to be considered, and has similar trade-offs compared to approach (i). In addition, samples with many unknowns that only have higher rank classifications can have an artificially short pairwise distance.

We scored how well the 150 pairwise dissimilarity measured represented the annotated biomes using PERMANOVA, and found that these groups are best represented by a dissimilarity measure based on an inverted Spearman's rank correlation coefficient ($0.5 - (\rho/2)$) at the taxonomic order rank while ignoring unknowns (**Supp. Fig. 6a-d)**. We thus use the Spearman's rank-based microbiome dissimilarity score to quantify SNB, while noting that another choice would not qualitatively affect our results, as social niche breadth scores based on six alternative ecological dissimilarity measures spanning the four different methods of dealing with unknowns showed a high correlation with the one we selected (**Supp. Fig. 6e**). In addition, we investigated the robustness of our results to our choice for the mean pairwise dissimilarity by comparing it to the median and third quartile, and found high correlations ($\rho = 0.977$ (p: 0.000) and $\rho = 0.967$ (p: 0.000), respectively; **Supp. Fig. 7**). In agreement with our premise that social niche breadth should reflect the cooccurrence of a taxon with other taxa, our SNB score is strongly negatively correlated with the fraction of shared taxa between samples ($\rho = -0.878$ (p: 0.000); **Supp. Fig. 8**).

Social niche breadth robustly reflects community heterogeneity

Robustness to sampling bias

To investigate the robustness of the SNB score to sampling bias, we further calculated social niche breadth for imaginary taxa (iSNB) that occur in all samples of an annotated biome. This showed a strong association between iSNB and the beta diversity of an annotated biome (**Supp. Fig. 9a**, **Supp. Fig. 4d**). Thus, taxa that are ubiquitously present in a very heterogeneous annotated biome would have a high SNB, while taxa that are ubiquitous in an annotated biome with a low heterogeneity would have a low SNB. Importantly, iSNB does not depend on the number of available samples for a given annotated biome (**Supp. Fig. 9b**). Random subsets of samples from single annotated biomes showed low variation in iSNB

(**Supp. Fig. 9c**, **Supp. Fig. 4e**), implying robustness of SNB to sporadically missed presence of a taxon in a sample, but standard deviation increased when the number of samples becomes very low (**Supp. Fig. 9c**, **Supp. Fig. 4e**). For this reason, we use caution when interpreting SNB of rare taxa, i.e. that are present in only a few samples, and exclude taxa that are present in less than 5 samples from our analyses. iSNB calculated for imaginary taxa that occur across two annotated biomes revealed low iSNB for highly similar annotated biomes like different human oral sites (**Supp. Fig. 10**, top left corner**).** In addition, even though presence in a single annotated biome with low beta diversity results in a low iSNB, presence in two different annotated biomes with low beta diversity still results in a high iSNB if they are very different from each other (**Supp. Fig. 10**).

For real microbial taxa, we observed a striking independence of SNB on the number of samples in which a taxon is found (**Fig. 2a-c**). Some moderately ubiquitous taxa are exclusively present in similar samples (low SNB), whereas many uncommon taxa are present in very different samples (high SNB). That some specialist taxa are still quite ubiquitous can partly be explained by the overrepresentation of some environments in our microbiome dataset, even though we selected a maximum of 1,000 samples per annotated biome (**Supp. Fig. 1b**). Further, we observed widely different SNB for taxa encountered in the same number of annotated biomes (**Fig. 2b**), pointing to differences in community dissimilarity between annotated biomes and heterogeneity within annotated biomes as discussed above. Nonetheless, many taxa that are found in only a few samples have a low SNB (**Supp. Fig. 11a**), indicating that the samples where they are found are similar in composition, and suggesting that rare taxa are often social specialists. The most cosmopolitan taxa are all social generalists (high SNB) (**Fig. 2a-c**), since they are present in many dissimilar samples.

We also calculated SNB based only on the subsets of samples belonging to all human and marine annotated biomes, and found good correlations with the SNB scores based on all samples($\rho = 0.546$ (p: 0.000) and $\rho = 0.662$ (p: 0.000), respectively; **Supp. Fig. 12**), implying that the sampling of annotated biomes does not strongly affect our calculated SNB values and suggesting that our general results would be qualitatively similar if different habitats were sampled.

Robustness to detection limit

The detection limit of taxa in environmental sequencing datasets is an important parameter that could influence SNB, as higher detection thresholds obscure our view of rare taxa[9] and decrease the number of samples and habitats in which taxa are found. To assess this effect, we calculated SNB with a ten-fold higher (1×10^{-3}) and ten-fold lower (1×10^{-5}) detection threshold than used for our main results (**Supp. Fig. 13**), and observed shifts in SNB as expected; overall, taxa become more specialist with a higher and more generalist with a lower detection threshold. Importantly, the list of taxa ranked by SNB was consistent (**Supp. Fig. 13c,g**), especially if uncommon taxa were excluded (**Supp. Fig. 13d,h**). In addition, exclusion of taxa that have very low relative abundance across samples does not change the distribution of SNB (**Supp. Fig. 11b**).

Robustness to experiment type

SNB is based on the presence of taxa in sequencing samples (22,518 in total) that are coming from different experiment types: amplicon (15,790 samples), metagenomic (1,097 samples), metatranscriptomic (13 samples), and 'unknown' (5,618 samples). The standardised taxonomic pipeline of MGnify allows for a comparison of these different experiment types which maximises the number of habitats on which the SNB score is based. The taxonomic classifications that we use are all based on the same SSU rRNA gene models that are queried in the samples irrespective of experiment type. We moreover selected analyses with at least 50,000 taxonomically annotated reads, ensuring that the targeted genes are there.

To further investigate the effect of including these different experiment types on SNB and our results, we first performed a PERMANOVA analysis with experiment type as the predefined groups, which showed very low R^2 values (**Supp. Fig. 6a-d**), implying a low impact of experiment type on the ecological clustering. To confirm that SNB does not depend considerably on the selection of experiment type, we calculated SNB for all taxa only based on the subsets of samples from the different experiment types (**Supp. Data File 3**). The taxa that were present in at least 5 samples in these subsets had similar SNB scores to their original SNB scores that are based on all samples, according to their rank order distribution: amplicon, 4,540 taxa, $\rho = 0.950$ (p: 0.000); metagenomic, 1,373 taxa, $\rho = 0.604$ (p: 0.000); metatranscriptomic, 530 taxa, $\rho = 0.677$ (p: 0.000); and 'unknown', 2,663 taxa, $\rho = 0.784$ (p: 0.000). Thus, taxa that are specialists or generalists in all samples also tend to be specialists or generalists in the experiment type subsets, respectively, justifying our decision to include these different data types in this global analysis.

Next, we investigated whether we would have obtained qualitatively different conclusions if we would have used only samples from one experiment type (**Supp. Fig. 14**). Importantly, our observations that generalist genera dominate local communities and have shorter doubling times than specialist genera are consistent across experiment type. Only the metagenomic samples do not show a higher variability of relative abundance across samples for social generalists than for social specialists but instead the opposite correlation, which may be a habitat specific observation as most metagenomic samples are from animal-associated environments (**Supp. Fig. 15**). The observations that social generalists have more diverse genomes than social specialists (measured in the standard deviation of their genome size) and a larger and more open pan genome are also consistently observed when basing our analyses on samples from single experiment types. In addition, clade age of social generalists is also older than that of social specialists across experiment type, with the exception of the FCA clade age in the metagenomic and metatranscriptomic subsets.

The most important observation that qualitatively differs when using only samples from a single experiment type as opposed to using all samples combined is the correlation between SNB and genome size. When using all samples we found no consistent relation between SNB and genome size. In contrast, the amplicon and metagenomic experiment types show a small positive correlation (i.e. social generalists have larger genomes than social specialists), whereas the metatranscriptomic and 'unknown' experiment types show a small negative correlation (i.e. social specialists have larger genomes than social generalists).

This discrepancy between experiment types can be largely attributed to differences in sampled habitats. The amplicon and metagenomic datasets contain relatively many animal-associated low alpha diversity samples, where social generalists tend to have larger genomes than social specialists (**Supp. Fig. 15**). In contrast, the 'unknown' dataset has relatively many free-living high alpha diversity samples, where social specialists tend to have larger genomes than social generalists (**Supp. Fig. 15**). This is consistent with the habitat specific relation between SNB and genome size that we observed earlier. However, most low alpha diversity samples in the 'unknown' dataset do not show a positive correlation between SNB and genome size (**Supp. Fig. 15**), in which they differ from the dataset based on all samples, and the metagenomic and amplicon samples. For example, the relatively low alpha diversity plant-associated samples of the 'unknown' dataset do not show a consistent correlation between SNB and genome size. This illustrates that the alpha diversity of a sample is only a proxy for habitat type and does not fully represent a taxon's niche. Lastly, all metatranscriptomic samples show a negative correlation between SNB and genome size, regardless of alpha diversity (**Supp. Fig. 15**). Although the low number of low alpha diversity samples in this subset prevents strong conclusions, comparing RNA-based samples (representing the active biomass) to DNA-based samples (representing all biomass) will be interesting for future research.

In conclusion, incorporation of different experiment types for the calculation of SNB does not fundamentally affect our general conclusions.

Together, the results presented in this section show that the SNB score is robust to the specific community dissimilarity measure, sampling bias, detection threshold, and experiment type, suggesting that our results represent a meaningful quantification of microbial niche range.

Supplementary Figures

Supplementary Figures 1-19.

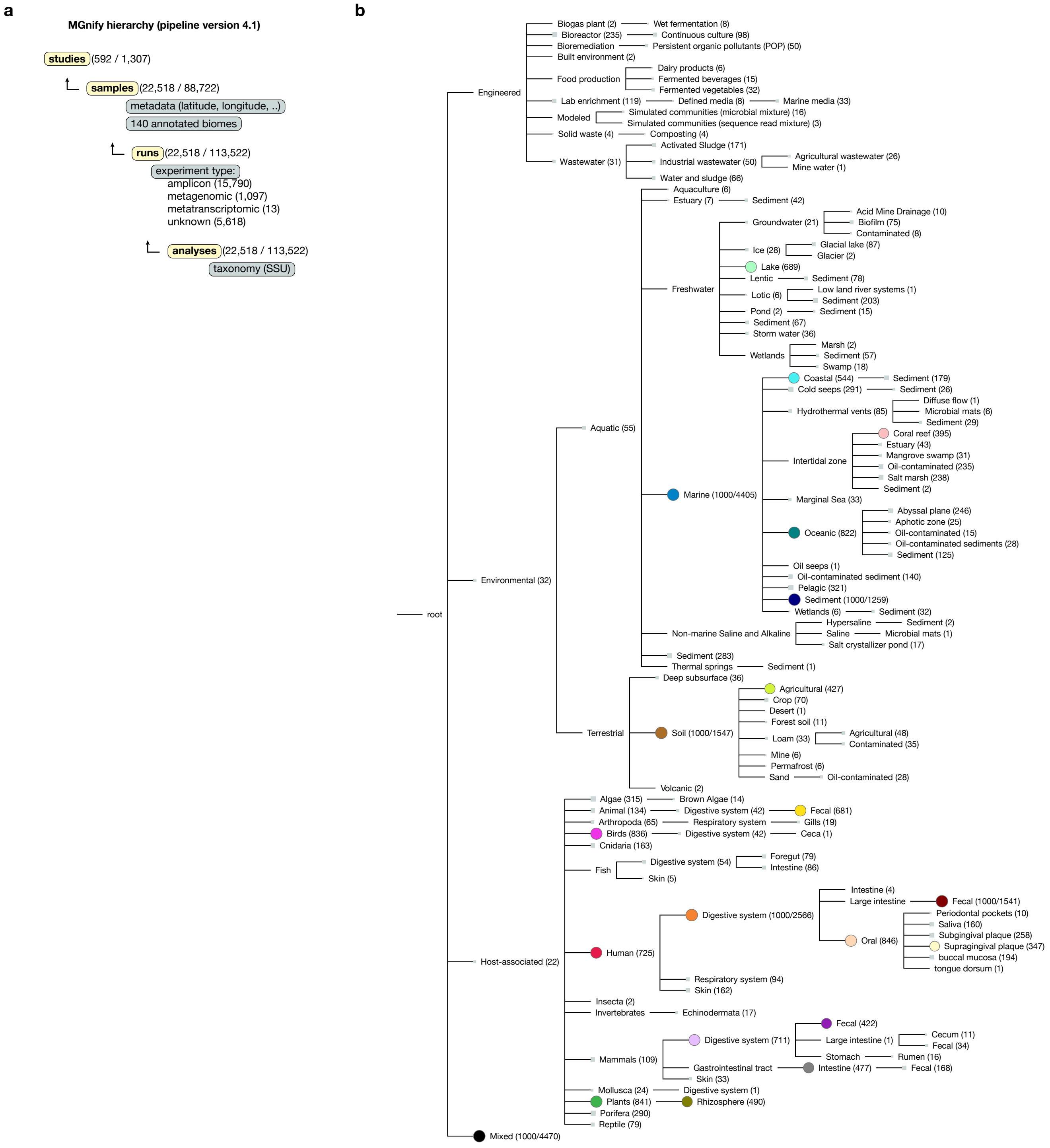

Supplementary Figure 1. Selection of samples and annotated biomes from the MGnify resource. (**a**) Studies in MGnify are divided into samples, which have associated runs and taxonomic analyses. Numbers within brackets indicate the number of selected instances out of the total in the resource that is annotated with pipeline version 4.1. A single taxonomic analysis on SSU level was picked per sample. For other selection criteria see **Methods**. (**b**) Hierarchical tree of annotated biomes. Numbers within brackets and size of markers indicates the number of samples from the annotated biome. If more than 1,000 samples met the selection criteria, 1,000 samples were picked at random and the total sample pool is indicated as the second number. Colours correspond to colour-coding of biomes in **Fig. 1**.

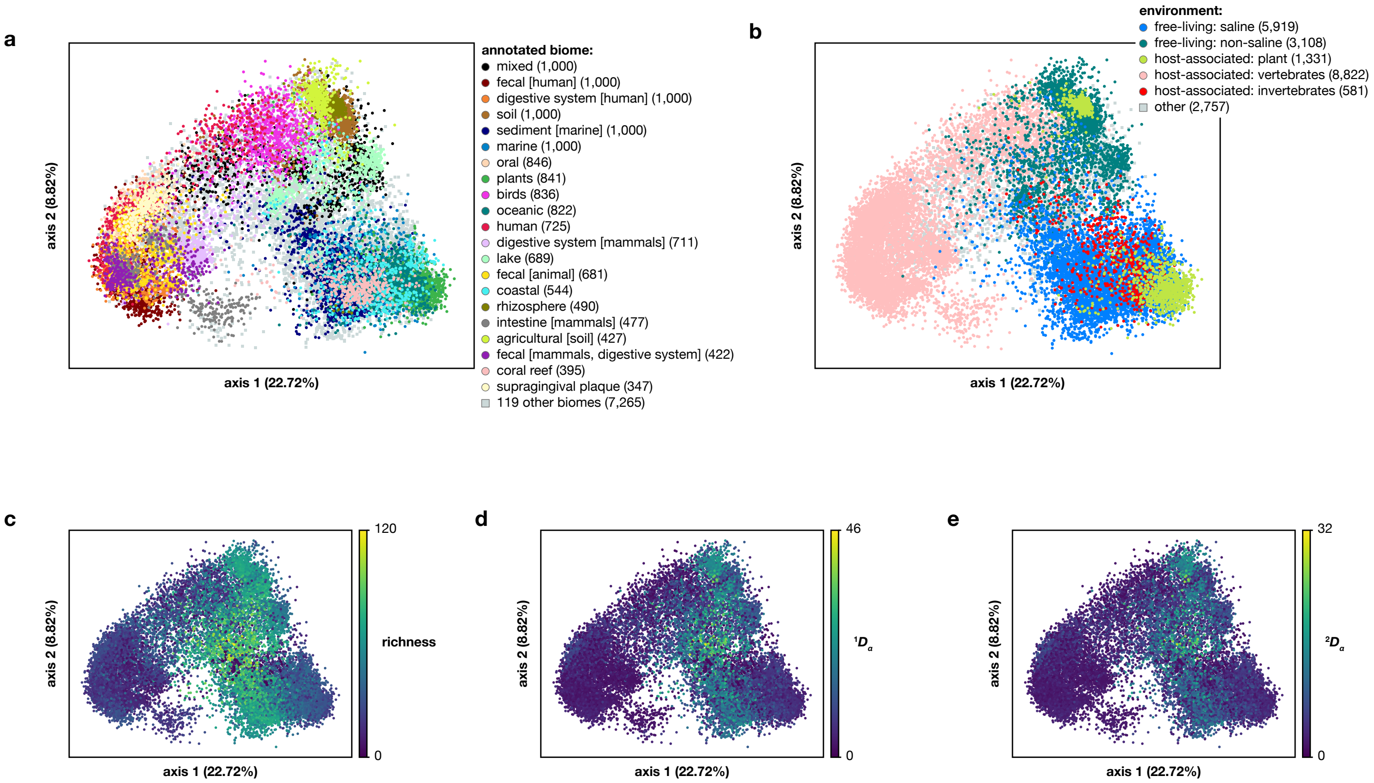

Supplementary Figure 2. PCoA visualisation of 22,518 microbiomes. (**a**) Samples from similar annotated biomes cluster together based on taxonomic profile, with the same ecological dissimilarity measure used as for SNB, namely Spearman's rank correlation coefficient $(0.5 - (\rho/2))$ of known taxa at rank order. (**b**) Samples are separated by host-association and salinity. Invertebrate-associated communities cluster together with free-living communities and not with vertebrate-associated communities. See **Supp. Fig. 3** for the division of annotated biomes in free-living and host-associated. See **Supp. Fig. 4a** for a t-SNE visualisation of the same data. (**c-e**) Alpha diversity of samples on the rank order for three different diversity measures, (c) zeroth order diversity (richness), (d) first order diversity $(e^{Shannon\ index})$, and (e) second order diversity (inverse Simpson index).

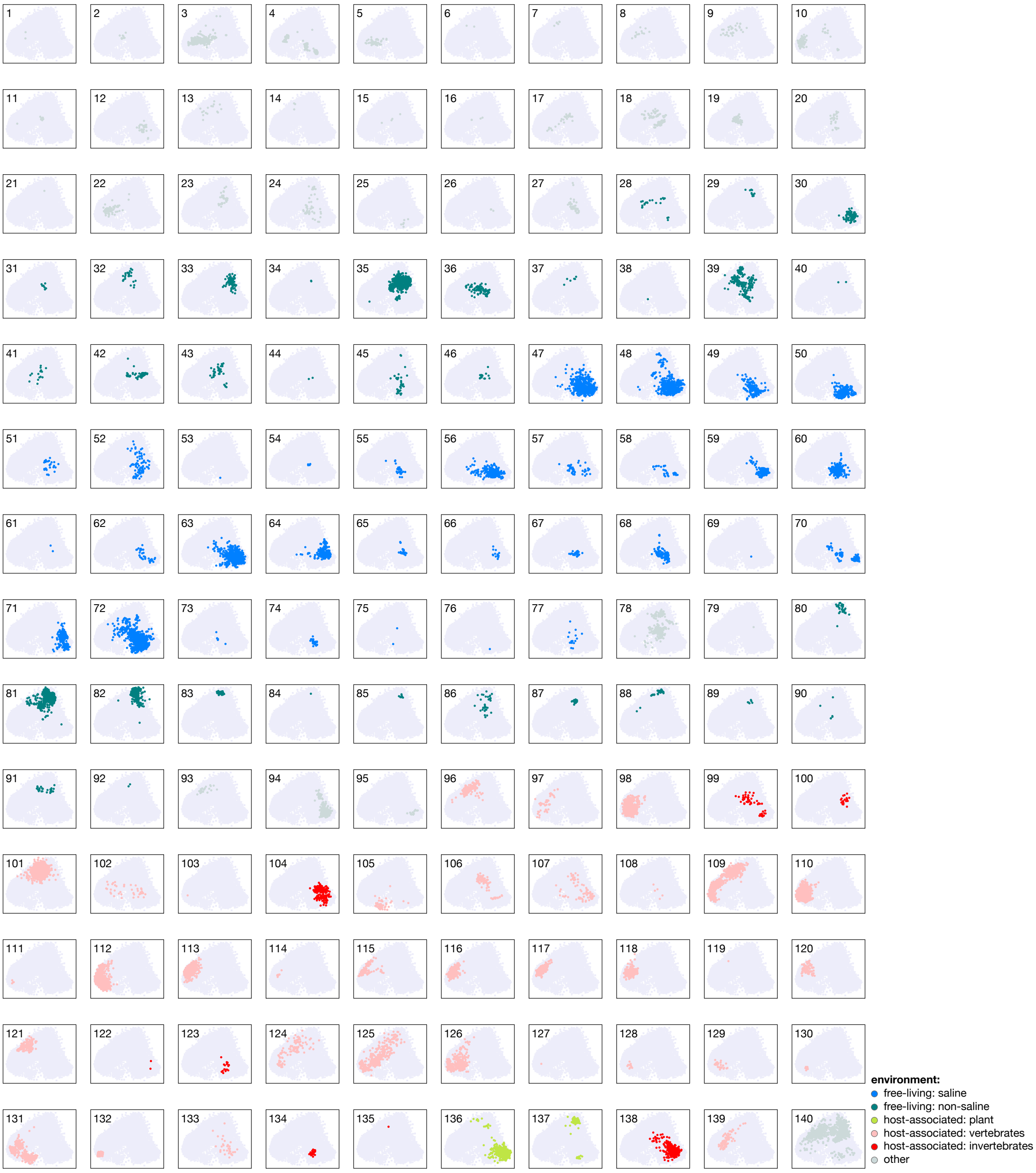

1. Engineered:Biogas plant (2)
2. Engineered:Biogas plant:Wet fermentation (8)
3. Engineered:Bioreactor (235)
4. Engineered:Bioreactor:Continuous culture (98)
5. Engineered:Bioremediation:Persistent organic pollutants (POP) (50)
6. Engineered:Built environment (2)
7. Engineered:Food production:Dairy products (6)
8. Engineered:Food production:Fermented beverages (15)
9. Engineered:Food production:Fermented vegetables (32)
10. Engineered:Lab enrichment (119)
11. Engineered:Lab enrichment:Defined media (8)
12. Engineered:Lab enrichment:Defined media:Marine media (33)
13. Engineered:Modeled:Simulated communities (microbial mixture) (16)
14. Engineered:Modeled:Simulated communities (sequence read mixture) (3)
15. Engineered:Solid waste (4)
16. Engineered:Solid waste:Composting (4)
17. Engineered:Wastewater (31)
18. Engineered:Wastewater:Activated Sludge (171)
19. Engineered:Wastewater:Industrial wastewater (50)
20. Engineered:Wastewater:Industrial wastewater:Agricultural wastewater (26)
21. Engineered:Wastewater:Industrial wastewater:Mine water (1)
22. Engineered:Wastewater:Water and sludge (66)
23. Environmental (32)
24. Environmental:Aquatic (55)
25. Environmental:Aquatic:Aquaculture (6)
26. Environmental:Aquatic:Estuary (7)
27. Environmental:Aquatic:Estuary:Sediment (42)
28. Environmental:Aquatic:Freshwater:Groundwater (21)
29. Environmental:Aquatic:Freshwater:Groundwater:Acid Mine Drainage (10)
30. Environmental:Aquatic:Freshwater:Groundwater:Biofilm (75)
31. Environmental:Aquatic:Freshwater:Groundwater:Contaminated (8)
32. Environmental:Aquatic:Freshwater:Ice (28)
33. Environmental:Aquatic:Freshwater:Ice:Glacial lake (87)
34. Environmental:Aquatic:Freshwater:Ice:Glacier (2)
35. Environmental:Aquatic:Freshwater:Lake (689)
36. Environmental:Aquatic:Freshwater:Lentic:Sediment (78)
37. Environmental:Aquatic:Freshwater:Lotic (6)
38. Environmental:Aquatic:Freshwater:Lotic:Low land river systems (1)
39. Environmental:Aquatic:Freshwater:Lotic:Sediment (203)
40. Environmental:Aquatic:Freshwater:Pond (2)
41. Environmental:Aquatic:Freshwater:Pond:Sediment (15)
42. Environmental:Aquatic:Freshwater:Sediment (67)
43. Environmental:Aquatic:Freshwater:Storm water (36)
44. Environmental:Aquatic:Freshwater:Wetlands:Marsh (2)
45. Environmental:Aquatic:Freshwater:Wetlands:Sediment (57)
46. Environmental:Aquatic:Freshwater:Wetlands:Swamp (18)
47. Environmental:Aquatic:Marine (1000)
48. Environmental:Aquatic:Marine:Coastal (544)
49. Environmental:Aquatic:Marine:Coastal:Sediment (179)
50. Environmental:Aquatic:Marine:Cold seeps (291)
51. Environmental:Aquatic:Marine:Cold seeps:Sediment (26)
52. Environmental:Aquatic:Marine:Hydrothermal vents (85)
53. Environmental:Aquatic:Marine:Hydrothermal vents:Diffuse flow (1)
54. Environmental:Aquatic:Marine:Hydrothermal vents:Microbial mats (6)
55. Environmental:Aquatic:Marine:Hydrothermal vents:Sediment (29)
56. Environmental:Aquatic:Marine:Intertidal zone:Coral reef (395)
57. Environmental:Aquatic:Marine:Intertidal zone:Estuary (43)
58. Environmental:Aquatic:Marine:Intertidal zone:Mangrove swamp (31)
59. Environmental:Aquatic:Marine:Intertidal zone:Oil-contaminated (235)
60. Environmental:Aquatic:Marine:Intertidal zone:Salt marsh (238)
61. Environmental:Aquatic:Marine:Intertidal zone:Sediment (2)
62. Environmental:Aquatic:Marine:Marginal Sea (33)
63. Environmental:Aquatic:Marine:Oceanic (822)
64. Environmental:Aquatic:Marine:Oceanic:Abyssal plane (246)
65. Environmental:Aquatic:Marine:Oceanic:Aphotic zone (25)
66. Environmental:Aquatic:Marine:Oceanic:Oil-contaminated (15)
67. Environmental:Aquatic:Marine:Oceanic:Oil-contaminated sediments (28)
68. Environmental:Aquatic:Marine:Oceanic:Sediment (125)
69. Environmental:Aquatic:Marine:Oil seeps (1)
70. Environmental:Aquatic:Marine:Oil-contaminated sediment (140)
71. Environmental:Aquatic:Marine:Pelagic (321)
72. Environmental:Aquatic:Marine:Sediment (1000)
73. Environmental:Aquatic:Marine:Wetlands (6)
74. Environmental:Aquatic:Marine:Wetlands:Sediment (32)
75. Environmental:Aquatic:Non-marine Saline and Alkaline:Hypersaline:Sediment (2)
76. Environmental:Aquatic:Non-marine Saline and Alkaline:Saline:Microbial mats (1)
77. Environmental:Aquatic:Non-marine Saline and Alkaline:Salt crystallizer pond (17)
78. Environmental:Aquatic:Sediment (283)
79. Environmental:Aquatic:Thermal springs:Sediment (1)
80. Environmental:Terrestrial:Deep subsurface (36)
81. Environmental:Terrestrial:Soil (1000)
82. Environmental:Terrestrial:Soil:Agricultural (427)
83. Environmental:Terrestrial:Soil:Crop (70)
84. Environmental:Terrestrial:Soil:Desert (1)
85. Environmental:Terrestrial:Soil:Forest soil (11)
86. Environmental:Terrestrial:Soil:Loam (33)
87. Environmental:Terrestrial:Soil:Loam:Agricultural (48)
88. Environmental:Terrestrial:Soil:Loam:Contaminated (35)
89. Environmental:Terrestrial:Soil:Mine (6)
90. Environmental:Terrestrial:Soil:Permafrost (6)
91. Environmental:Terrestrial:Soil:Sand:Oil-contaminated (28)
92. Environmental:Terrestrial:Volcanic (2)
93. Host-associated (22)
94. Host-associated:Algae (315)
95. Host-associated:Algae:Brown Algae (14)
96. Host-associated:Animal (134)
97. Host-associated:Animal:Digestive system (42)
98. Host-associated:Animal:Digestive system:Fecal (681)
99. Host-associated:Arthropoda (65)
100. Host-associated:Arthropoda:Respiratory system:Gills (19)
101. Host-associated:Birds (836)
102. Host-associated:Birds:Digestive system (42)
103. Host-associated:Birds:Digestive system:Ceca (1)
104. Host-associated:Cnidaria (163)
105. Host-associated:Fish:Digestive system (54)
106. Host-associated:Fish:Digestive system:Foregut (79)
107. Host-associated:Fish:Digestive system:Intestine (86)
108. Host-associated:Fish:Skin (5)
109. Host-associated:Human (725)
110. Host-associated:Human:Digestive system (1000)
111. Host-associated:Human:Digestive system:Intestine (4)
112. Host-associated:Human:Digestive system:Large intestine:Fecal (1000)
113. Host-associated:Human:Digestive system:Oral (846)
114. Host-associated:Human:Digestive system:Oral:Periodontal pockets (10)
115. Host-associated:Human:Digestive system:Oral:Saliva (160)
116. Host-associated:Human:Digestive system:Oral:Subgingival plaque (258)
117. Host-associated:Human:Digestive system:Oral:Supragingival plaque (347)
118. Host-associated:Human:Digestive system:Oral:buccal mucosa (194)
119. Host-associated:Human:Digestive system:Oral:tongue dorsum (1)
120. Host-associated:Human:Respiratory system (94)
121. Host-associated:Human:Skin (162)
122. Host-associated:Insecta (2)
123. Host-associated:Invertebrates:Echinodermata (17)
124. Host-associated:Mammals (109)
125. Host-associated:Mammals:Digestive system (711)
126. Host-associated:Mammals:Digestive system:Fecal (422)
127. Host-associated:Mammals:Digestive system:Large intestine (1)
128. Host-associated:Mammals:Digestive system:Large intestine:Cecum (11)
129. Host-associated:Mammals:Digestive system:Large intestine:Fecal (34)
130. Host-associated:Mammals:Digestive system:Stomach:Rumen (16)
131. Host-associated:Mammals:Gastrointestinal tract:Intestine (477)
132. Host-associated:Mammals:Gastrointestinal tract:Intestine:Fecal (168)
133. Host-associated:Mammals:Skin (33)
134. Host-associated:Mollusca (24)
135. Host-associated:Mollusca:Digestive system (1)
136. Host-associated:Plants (841)
137. Host-associated:Plants:Rhizosphere (490)
138. Host-associated:Porifera (290)
139. Host-associated:Reptile (79)
140. Mixed (1000)

Supplementary Figure 3. Location of all annotated biomes on the PCoA. Annotated biomes are coloured according to their environment type, free-living or host-associated. The 'Animal' biomes contain samples from frog, iguana, cats, dog, pigeon, fish, rat, cow, pig, mouse, poultry, and mammalia-associated habitat (**Supp. Data File 1**), and are thus included in the vertebrate environment type.

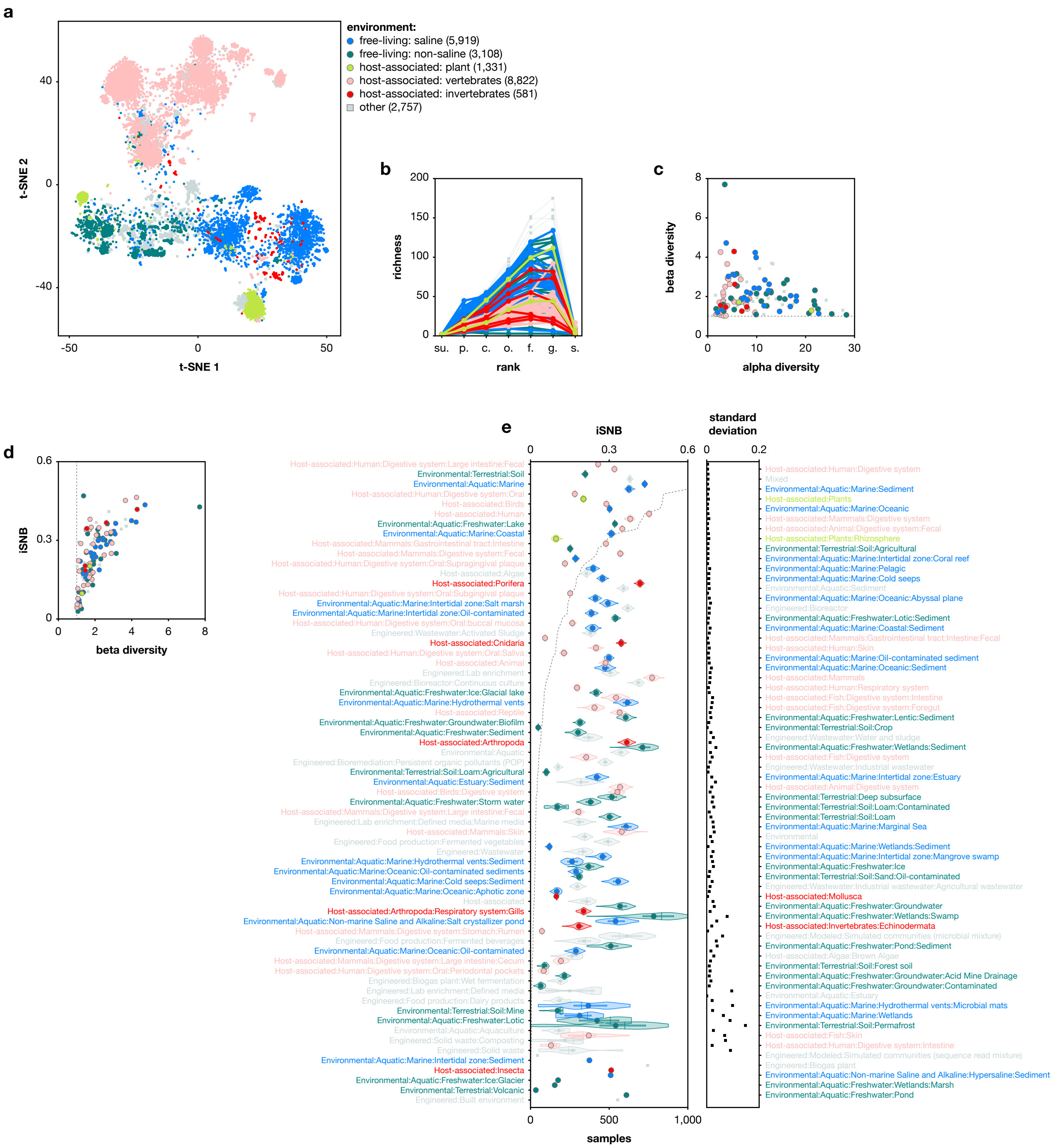

Supplementary Figure 4. Remake of some figures colour-coded according to environment type. (**a**) Idem to **Fig. 1d**. (**b**) Idem to **Fig. 1e**. (**c**) Idem to **Fig. 1f.** (**d**) Idem to **Supp. Fig. 9a**. (**e**) Idem to **Supp. Fig. 9c.** Figures are identical except for the colour-coding.

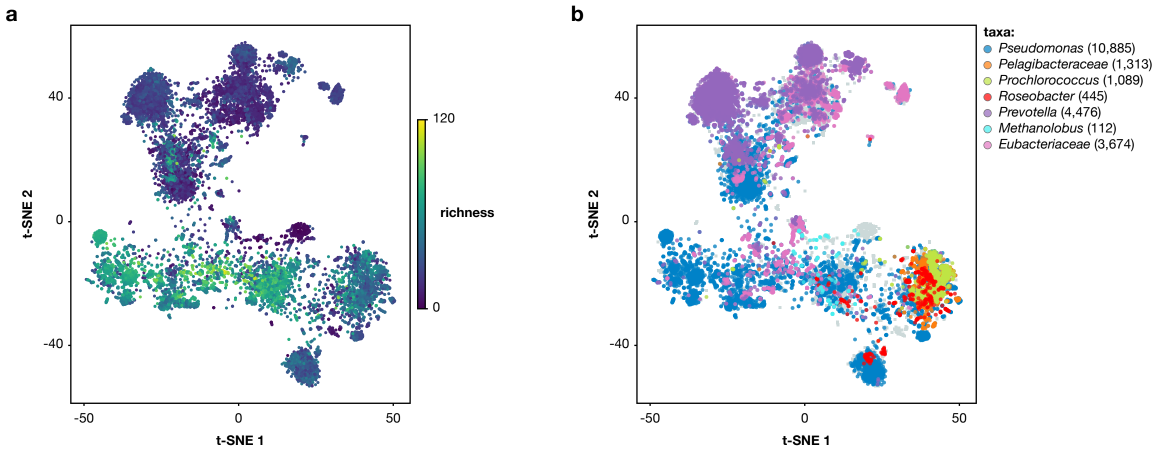

Supplementary Figure 5. Richness of samples and presence of some taxa visualised on the t-SNE of Figure 1d. (**a**) Zeroth order alpha diversity (richness) on the rank order of samples. (**b**) Presence of some microbial taxa.

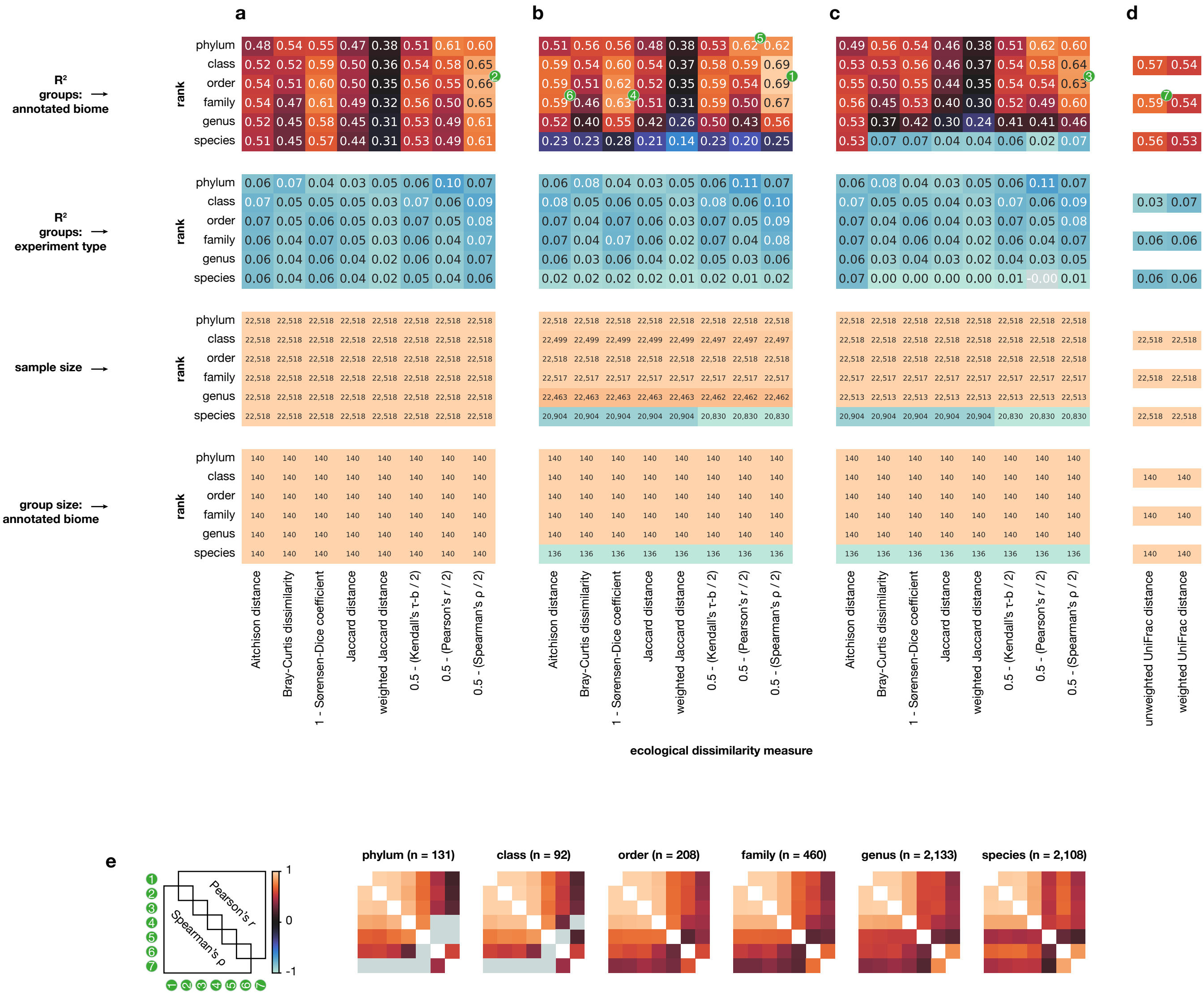

Supplementary Figure 6. Selection of ecological dissimilarity measure. PERMANOVA results with annotated biomes or experiment type (amplicon, metagenomic, metatranscriptomic, or 'unknown') as predefined groups (see **Supp. Fig. 1**). Dissimilarity between any two samples was calculated for ten different dissimilarity measures and three different methods for dealing with unknowns. See **Methods** for a description of these methods. (**a**) Approach i. (**b**)Approach ii. (**c**)Approach iii. (**d**) Unifrac distances. The sample size and group size for the annotated biomes analyses are indicated which can be smaller than the total number of samples or annotated biomes in the dataset, respectively, if samples do not contain enough taxa for a pairwise comparison. The group size for the experiment type analyses was four. (**e**) Correlations between niche breadth of taxa calculated with 7 different ecological dissimilarity measures (green badges in panels a-d) at different taxonomic ranks. Values in panels a-e that are not significant (p > 0.05) are coloured grey.

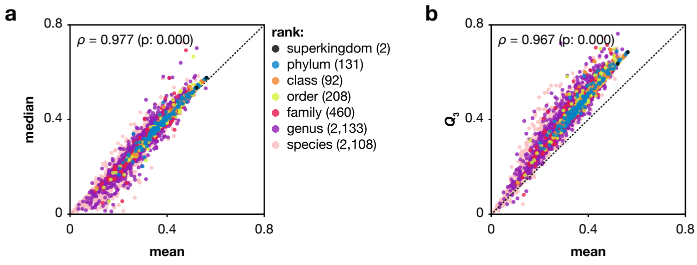

Supplementary Figure 7. SNB is robust against the choice for mean pairwise distance between the samples containing a taxon. (**a**) SNB of taxa calculated with mean versus median pairwise distance. (**b**) SNB of taxa calculated with mean versus the 75th percentile pairwise distance. Numbers within brackets behind ranks indicate number of taxa. Text in the top of the panels are Spearman's rank correlation coefficient and associated p-value calculated for all taxa.

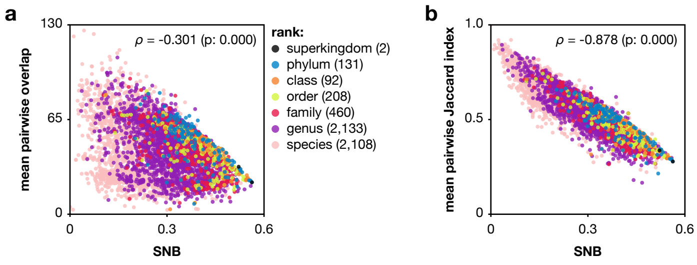

Supplementary Figure 8. SNB correlates with the fraction of overlapping taxa between the samples containing a taxon. (**a**) SNB of taxa versus mean absolute pairwise overlap. (**b**) SNB of taxa versus mean relative pairwise overlap. Overlap was calculated on the taxonomic rank order. Numbers within brackets behind ranks indicate number of taxa. Text in the top of the panels show Spearman's rank correlation coefficient and associated p-value calculated for all taxa.

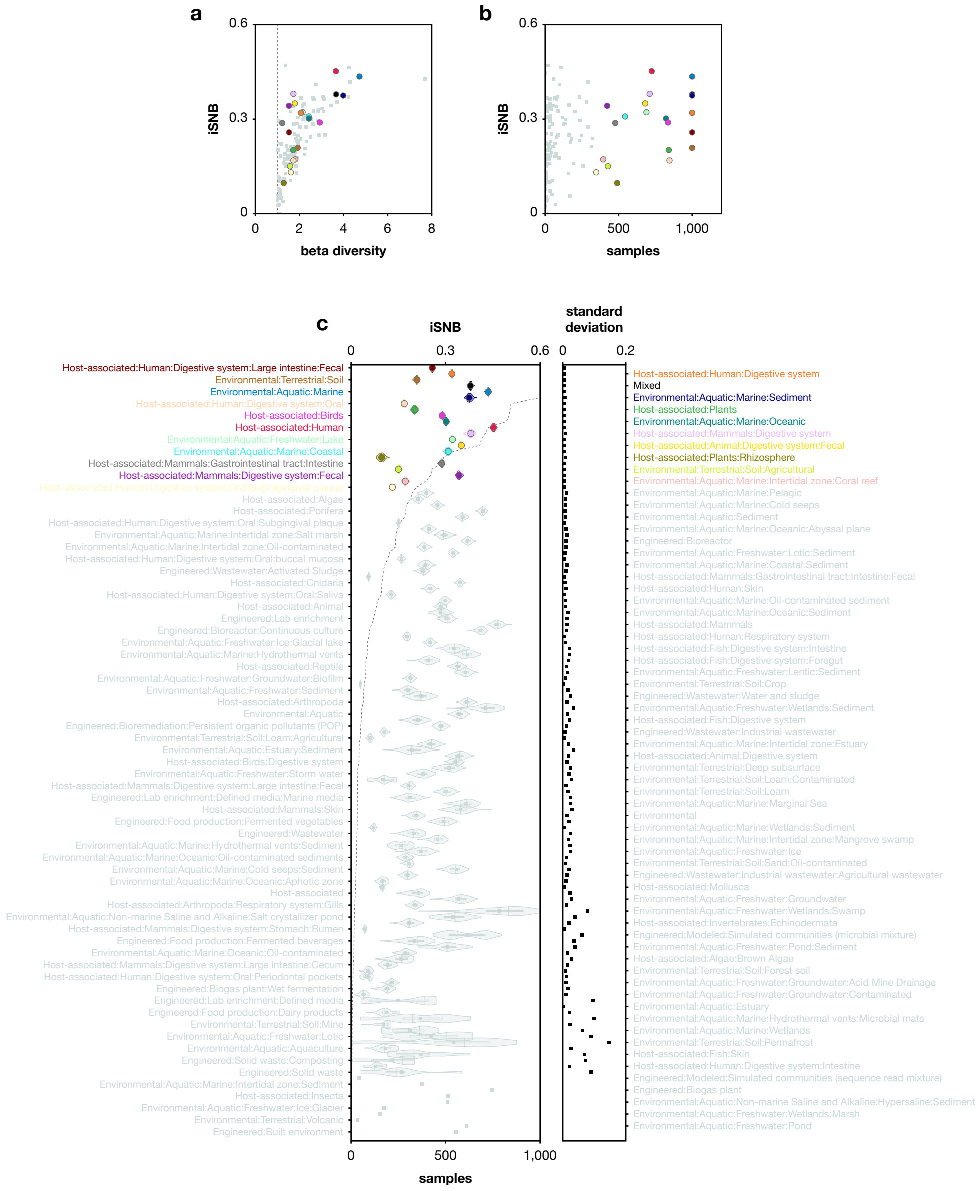

Supplementary Figure 9. SNB reflects beta diversity and is largely independent of the number of samples in which a taxon is detected. (**a**) Beta diversity of annotated biomes versus niche breadth for imaginary taxa (iSNB) that are found in all samples of an annotated biome. (**b**) Number of samples of annotated biomes versus their iSNB. (**c**) iSNB calculated for hypothetical taxa that are found in all samples of an annotated biome (dots) and 100 times randomly picked 50% of the samples (violins). Lines within violins show interquartile range and median. Annotated biomes are sorted according to number of samples (dashed line). The standard deviation of iSNB of the 100 randomly picked samples is depicted on the right.

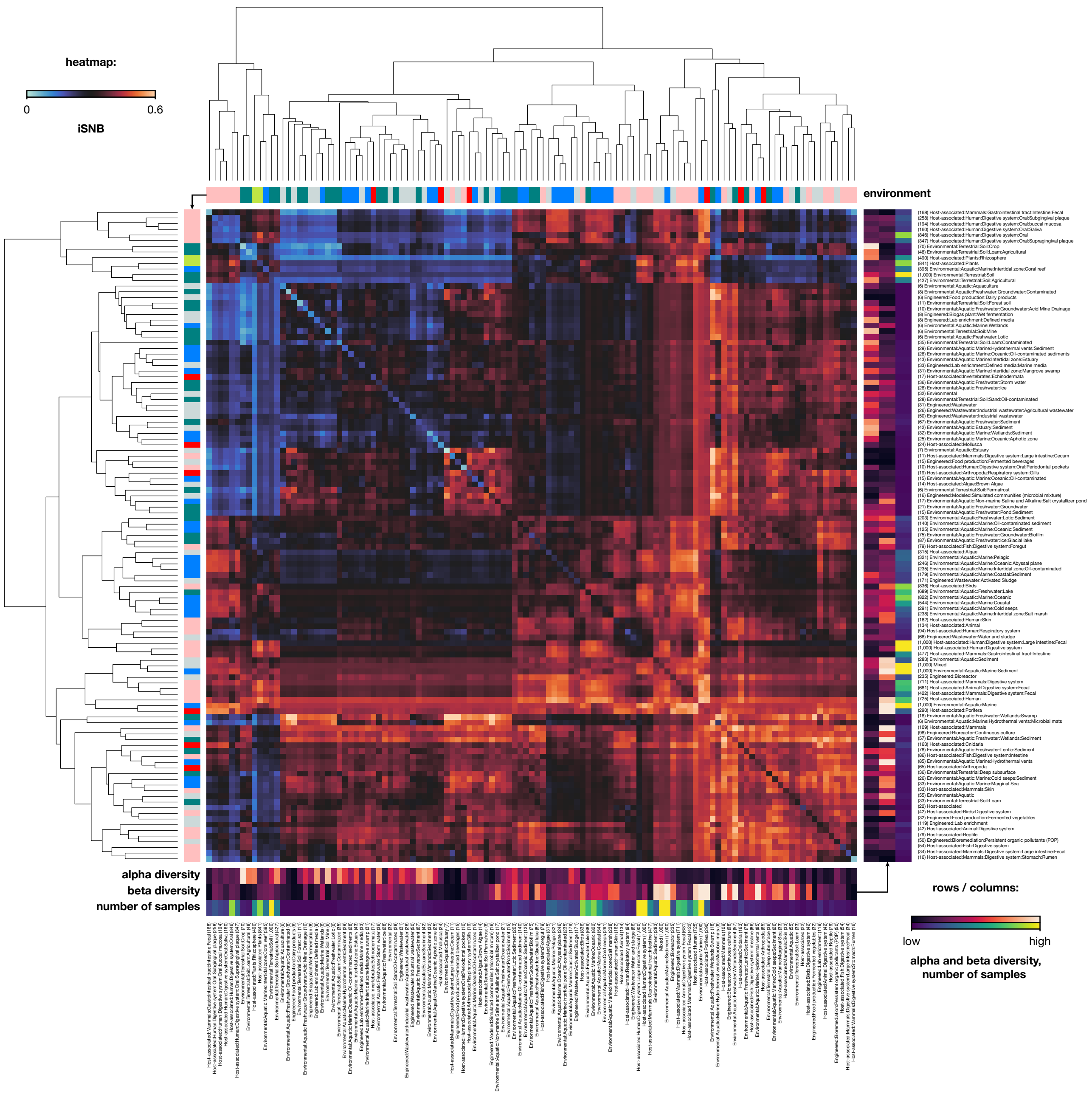

Supplementary Figure 10. Niche breadth of hypothetical taxa (iSNB) that are present in all samples of all combinations of two annotated biomes. Hierarchical clustering of the heatmap is based on euclidean distance and the UPGMA algorithm. Alpha and beta diversity and number of samples are indicated with colour-coding along the axes. Number of samples are also indicated in the labels within brackets. The environment type colour-coding corresponds to **Supp. Fig. 3**.

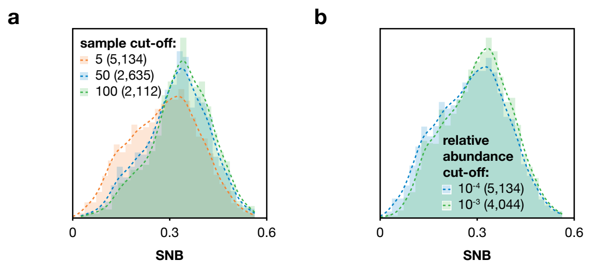

Supplementary Figure 11. Response of the overall distribution of SNB scores to different parameter cut-offs in our pipeline. (**a**) The minimum number of samples in which a taxon must be found. (**b**) The minimum relative abundance that must be reached by a taxon in at least 1 sample. Numbers within brackets indicate number of taxa.

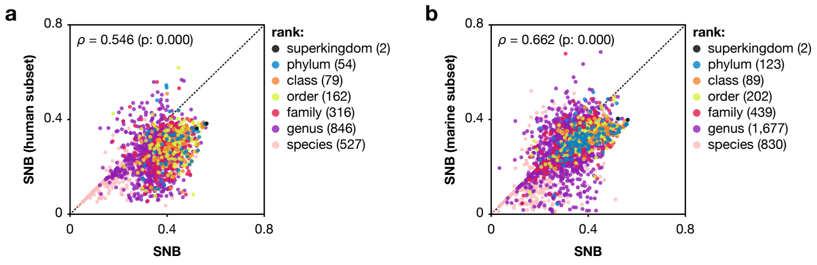

Supplementary Figure 12. SNB is relatively invariant to environmental scale. SNB based on all samples versus SNB based on the hierarchical subsets of (**a**) human annotated biomes and (**b**) marine annotated biomes. See **Supp. Fig. 1b** for which annotated biomes are included in the 'Human' and 'Marine' hierarchical subsets. Numbers within brackets behind ranks indicate number of taxa. Text in the top of the panels are Spearman's rank correlation coefficient and associated p-value calculated for all taxa.

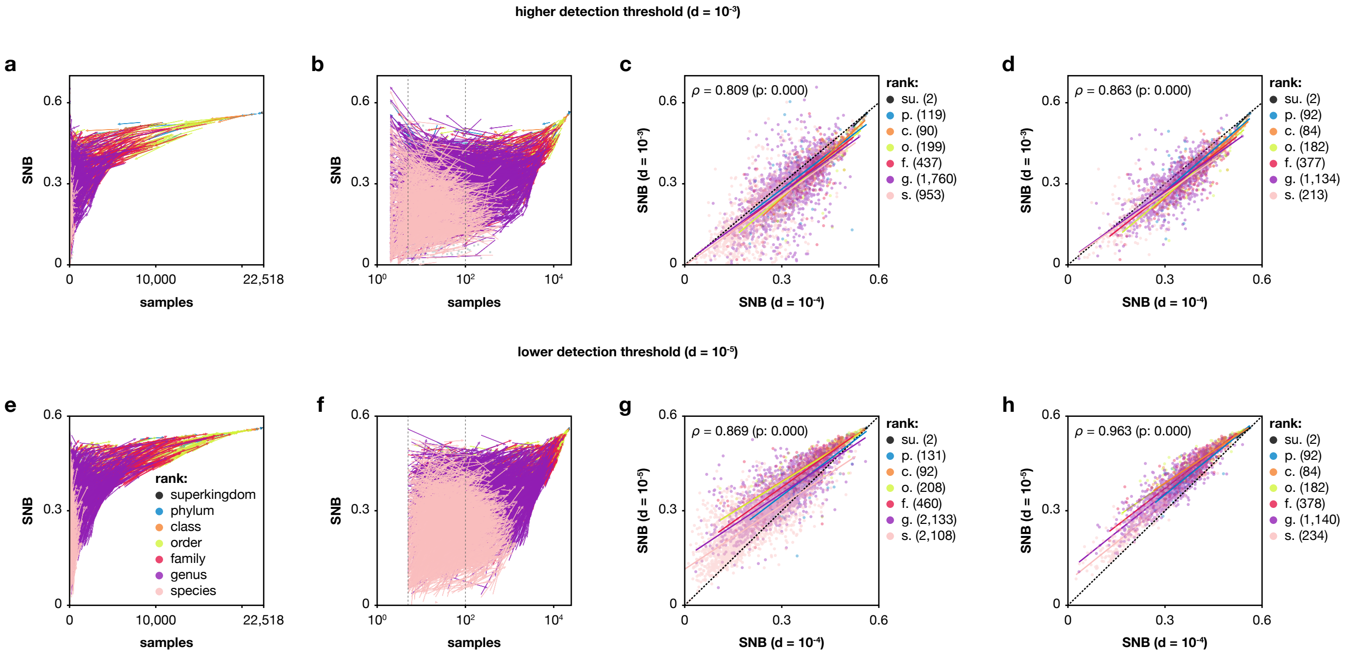

Supplementary Figure 13. Effect of the detection threshold on SNB. (**a,e**) Arrows show the change in number of samples and SNB due to a higher and lower detection threshold. (**b,f**) The same figure but with a logarithmic x-axis. Dashed lines indicate a presence in 5 samples which is the default cut-off for taxa to be included in most analyses in this study, and a presence in 100 samples which is the cut-off for panels d and h. (**c,g**) SNB with default detection threshold versus SNB with a higher or lower detection threshold. Coloured lines are linear regression lines for different taxonomic ranks. Taxa that had a presence in less than 5 samples with a higher detection threshold (see panel b) are included. (**d,h**) The same figure but with taxa that are present in less than 100 samples removed (dashed lines in panels b and f). Numbers within brackets behind ranks indicate number of taxa. Text in the top of panels c,d,g,h are Spearman's rank correlation coefficient and associated p-value calculated for all taxa.

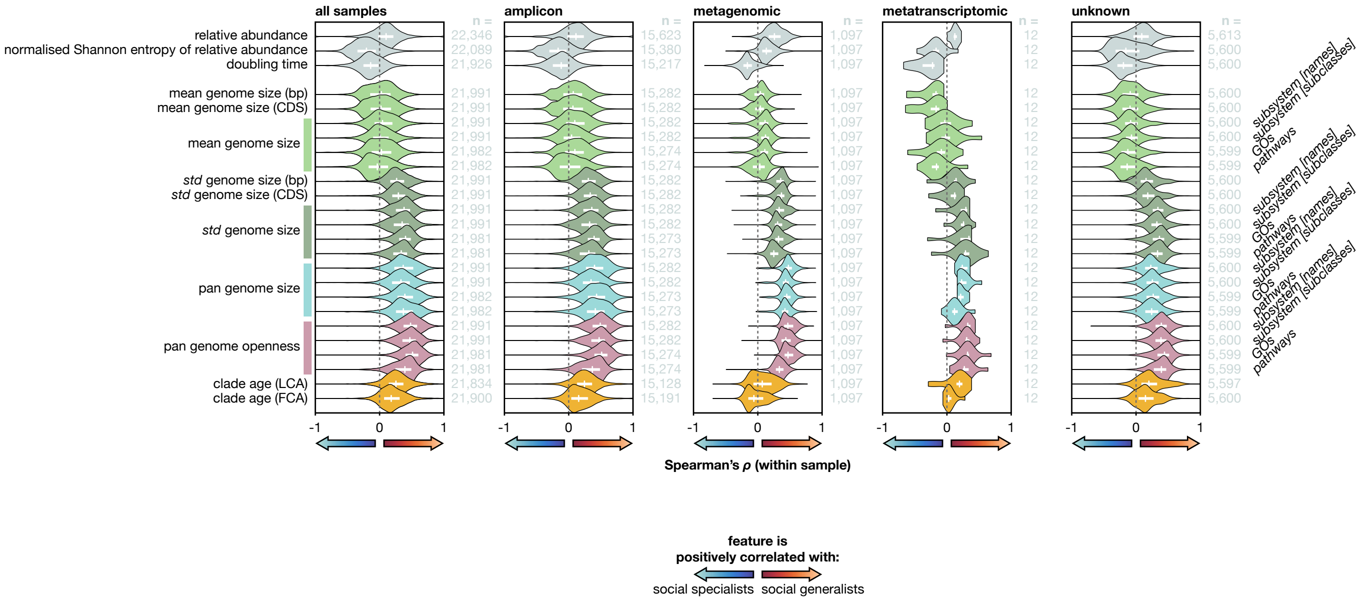

Supplementary Figure 14. Ecological and genomic features correlated with SNB if only samples from a single experiment type are used. Spearman's rank correlation coefficient (ρ) within samples between SNB and features related to local dominance and genomic features on the rank genus. The first panel is a repetition of **Fig. 3**, the other panels are what these results would have looked like if SNB and the shown correlations are only based on samples from that specific experiment type. Numbers to the right of violins show sample size, lines within violins show interquartile range and median. Source data of the figure are available in **Supp. Data File 6**.

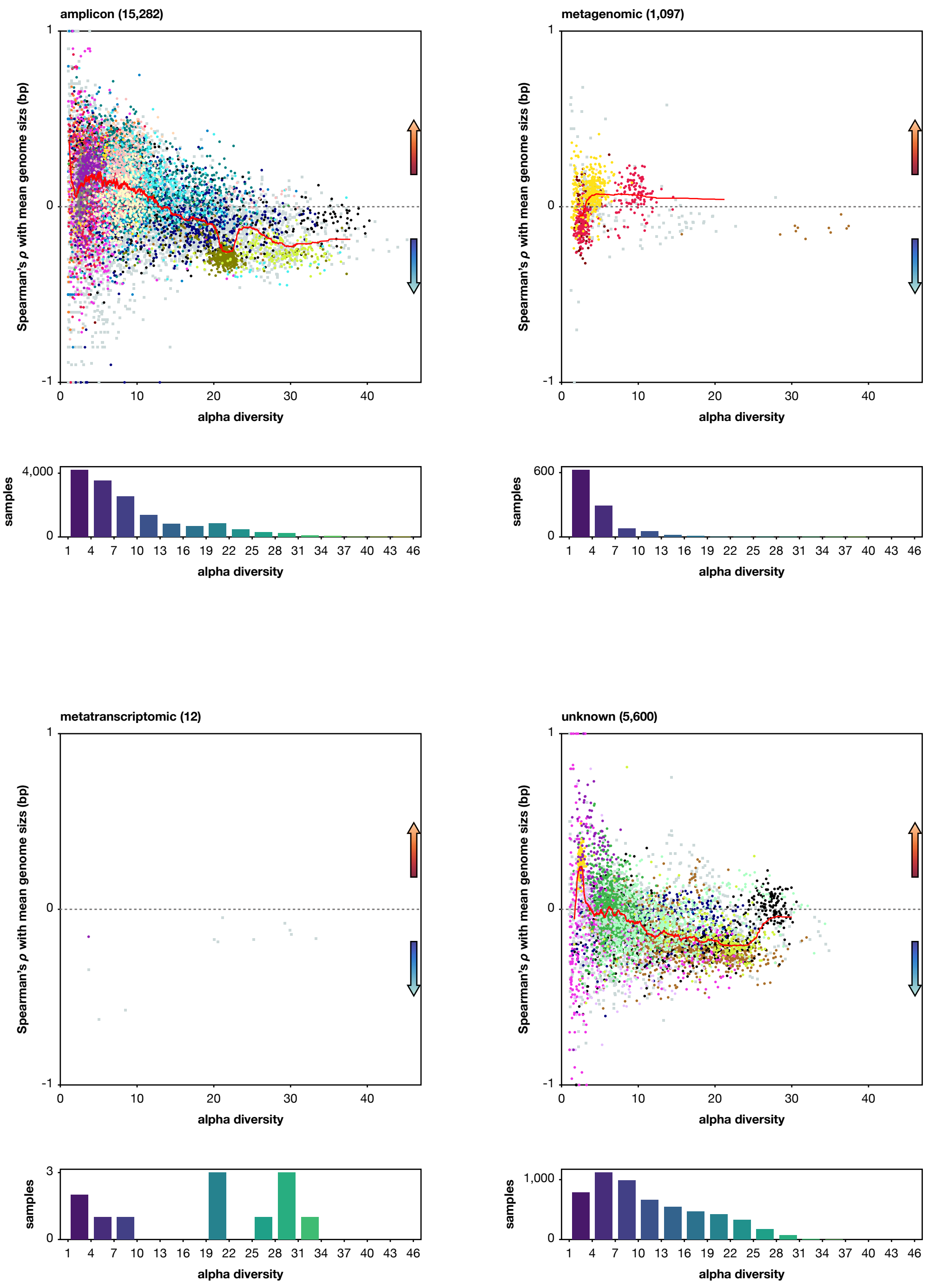

Supplementary Figure 15. Contrasting genomic niche range strategies if only samples from a single experiment type are used. The panels depict what **Fig. 4b,c** would have looked like if SNB is only based on samples from the specific experiment type. Numbers within brackets show the number of samples that are included in that experiment type, which can be lower than their total number of samples because a correlation coefficient can not be calculated when the number of genera in a sample is ≤2, and samples with no classifications at order rank (two in total) do not have an alpha diversity definition. Source data of the figure are available in **Supp. Data File 6.**

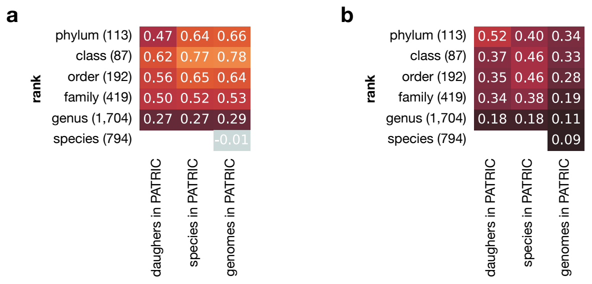

Supplementary Figure 16. Correlation between SNB of a taxon and its number of subtaxa in the PATRIC database. Daughters are the number of taxa one rank below the current rank. (**a**) Spearman's rank correlation coefficient, and (**b**) Pearson correlation coefficient. The correlation with number of genomes in PATRIC on the species rank in panel a was not significant ($p > 0.05$) and is coloured grey. Numbers within brackets indicate number of taxa.

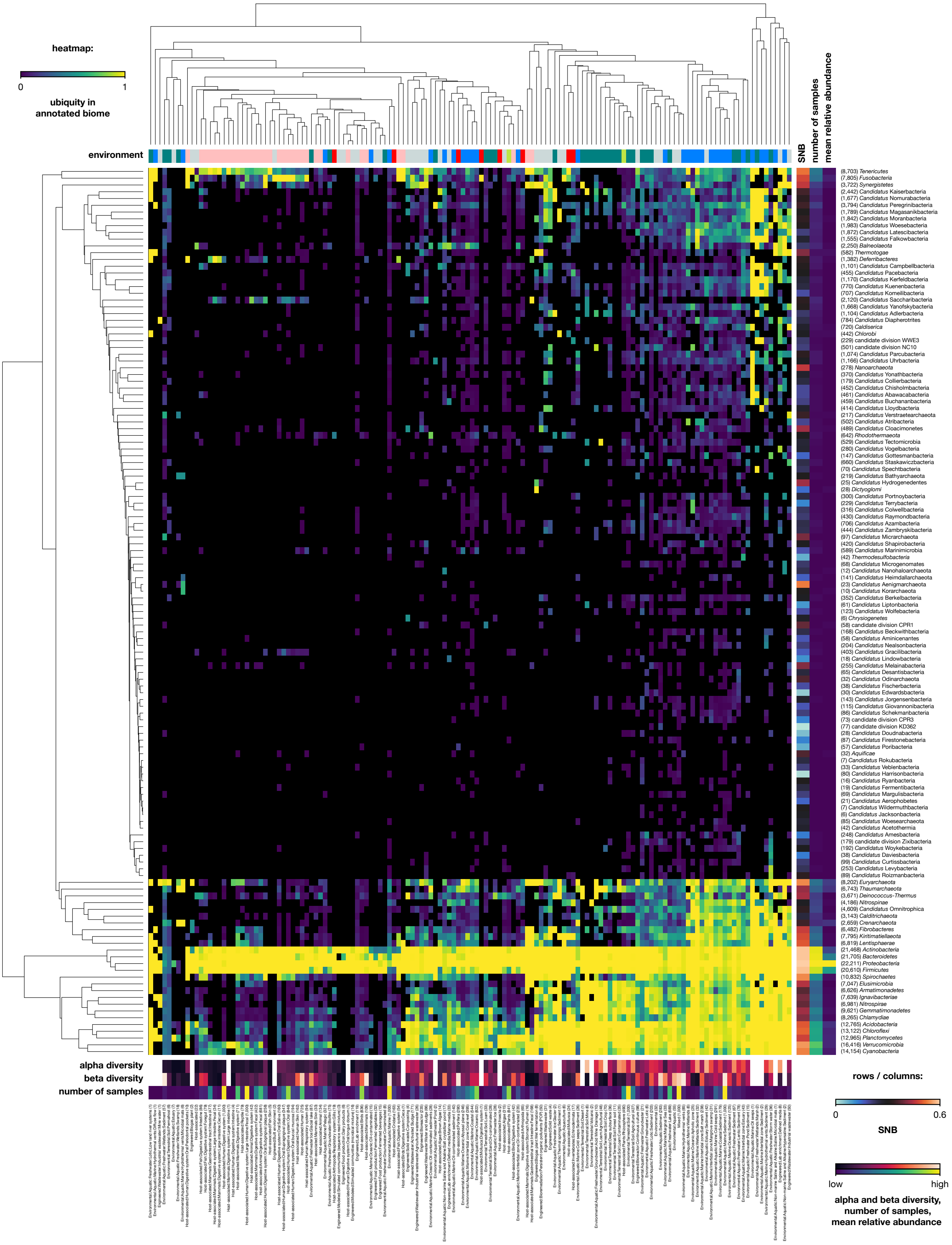

Supplementary Figure 17. Distribution of phyla in annotated biomes. Heatmap shows the fraction of samples of an annotated biome in which the phylum is found. Hierarchical clustering of heatmap is based on Euclidean distance and the UPGMA algorithm. Alpha and beta diversity, and number of samples of biomes are indicated with colour-coding along the x-axis. SNB, number of samples, and mean relative abundance of a phylum are indicated with colour-coding along the y-axes. Number of samples are also indicated in the labels within brackets. The environment type colour-coding corresponds to **Supp. Fig. 3**.

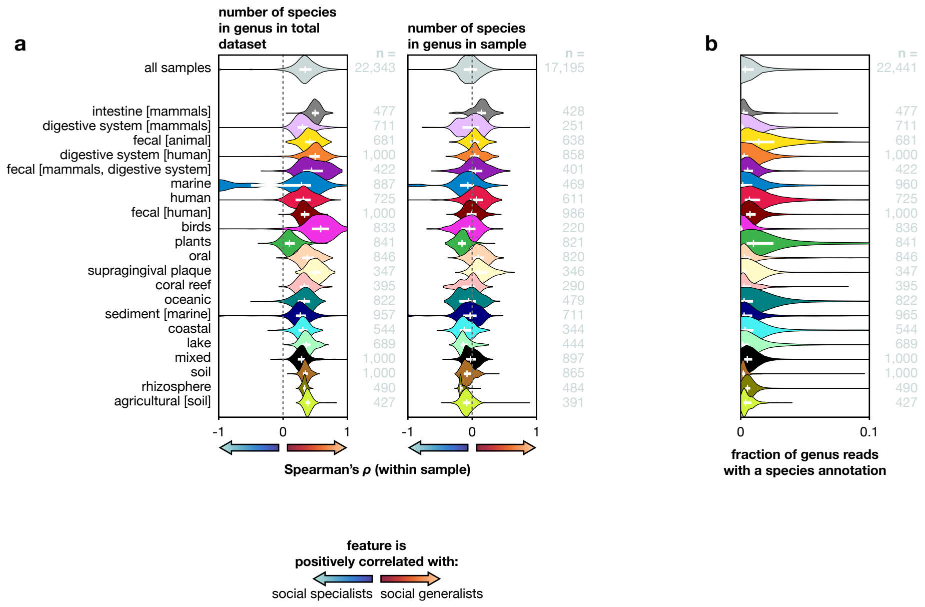

Supplementary Figure 18. Number of species in genera in the MGnify dataset. (**a**) Spearman's rank correlation coefficient (ρ) within samples between SNB and features related to the number of species in a genus. The number of species in the total dataset are all species that have an absolute abundance of at least 5 reads in one of the samples. The number of species in a sample are all species that have an absolute abundance of at least 5 reads in the sample. When at least 5 reads that map to a genus in a sample are not accounted for by its species, an extra 'unknown' species was added. In both plots, when a genus did not have any species annotation its number of species was set to 1. Violins depict the distribution of ρ across all samples or those from the annotated biomes with the most samples. Source data are available in **Supp. Data File 6.** (**b**) Fraction of genus reads that have a species annotation within the sample. Numbers to the right of violins show sample size, lines within violins in panel a and b show interquartile range and median.

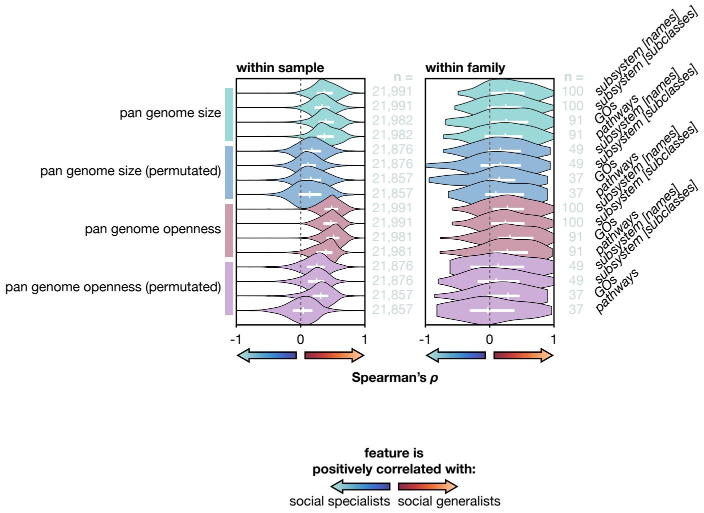

Supplementary Figure 19. Correlations between SNB and pan genome size and pan genome openness do not depend on a higher number of species in generalists. Spearman's rank correlation coefficient ρ between SNB and pan genomic features on the rank genus within samples and within families. Violins depict the distribution of ρ across all communities or across all families with at least 5 genera. Measures are in number of unique functions for the functional universe on the right. Measures with '(permutated)' in the name are based on the mean of 1,000 randomly picked subsets of 3 species from the genus and thus correct for the high number of species in some genera. Genera with less than 3 species were excluded from these analyses. The non-permutated violins are identical to the violins in **Fig. 3**. Genome size estimates for a genus are based on the genome size of its species, which is defined as the majority set of functions of all strains for the functional universe measures. Pan genome openness is total pan genome size divided by mean genome size. Numbers to the right of violins show sample size, lines within violins show interquartile range and median. Source data of the figure are available in **Supp. Data File 6** and **Supp. Data File 7**.

References

1. Mitchell, A. L. *et al.* MGnify: the microbiome analysis resource in 2020. *Nucleic Acids Res* **48**, D570–D578 (2020).

2. Lozupone, C. A. & Knight, R. Global patterns in bacterial diversity. *Proc National Acad Sci* **104**, 11436–11440 (2007).

3. Ley, R. E., Lozupone, C. A., Hamady, M., Knight, R. & Gordon, J. I. Worlds within worlds: evolution of the vertebrate gut microbiota. *Nat Rev Microbiol* **6**, 776–788 (2008).

4. Thompson, L. R. *et al.* A communal catalogue reveals Earth's multiscale microbial diversity. *Nature* **551**, 457–463 (2017).

5. Auguet, J.-C., Barberan, A. & Casamayor, E. O. Global ecological patterns in uncultured Archaea. *Isme J* **4**, 182–190 (2010).

6. Aitchison, J., Barceló-Vidal, C., Martín-Fernández, J. A. & Pawlowsky-Glahn, V. Logratio Analysis and Compositional Distance. *Math Geol* **32**, 271–275 (2000).

7. Gloor, G. B., Macklaim, J. M., Pawlowsky-Glahn, V. & Egozcue, J. J. Microbiome Datasets Are Compositional: And This Is Not Optional. *Front Microbiol* **8**, 2224 (2017).

8. Lozupone, C., Lladser, M. E., Knights, D., Stombaugh, J. & Knight, R. UniFrac: an effective distance metric for microbial community comparison. *Isme J* **5**, 169–172 (2011).

9. Lynch, M. D. J. & Neufeld, J. D. Ecology and exploration of the rare biosphere. *Nat Rev Microbiol* **13**, 217–229 (2015).

10. Schloss, P. D., Girard, R. A., Martin, T., Edwards, J. & Thrash, J. C. Status of the Archaeal and Bacterial Census: an Update. *Mbio* **7**, e00201-16 (2016).

11. Garcia-Pichel, F., Zehr, J. P., Bhattacharya, D. & Pakrasi, H. B. What's in a name? The case of cyanobacteria. *J Phycol* **56**, 1–5 (2020).

12. Takeuchi, M. *et al.* Methyloceanibacter caenitepidi gen. nov., sp. nov., a facultatively methylotrophic bacterium isolated from marine sediments near a hydrothermal vent. *Int J Syst Evol Micr* **64**, 462–468 (2014).
