# Supplementary material for: A social niche breadth score reveals niche range strategies of generalists and specialists
Source: Nat Ecol Evol. 2023 Apr 3;7(5):768–81. doi: 10.1038/s41559-023-02027-7 (PMC10172124; doi:10.1038/s41559-023-02027-7)
Supplement: Supplementary file 2 — Reporting Summary [file 41559_2023_2027_MOESM2_ESM.pdf]

nature portfolio

Corresponding author(s): Bas E. Dutilh

Last updated by author(s): Feb 21, 2023

Reporting Summary

Nature Portfolio wishes to improve the reproducibility of the work that we publish. This form provides structure for consistency and transparency in reporting. For further information on Nature Portfolio policies, see our Editorial Policies and the Editorial Policy Checklist.

Statistics

For all statistical analyses, confirm that the following items are present in the figure legend, table legend, main text, or Methods section.

n/a	Confirmed	
☐	☒	The exact sample size (*n*) for each experimental group/condition, given as a discrete number and unit of measurement
☐	☒	A statement on whether measurements were taken from distinct samples or whether the same sample was measured repeatedly
☐	☒	The statistical test(s) used AND whether they are one- or two-sided<br>*Only common tests should be described solely by name; describe more complex techniques in the Methods section.*
☐	☒	A description of all covariates tested
☐	☒	A description of any assumptions or corrections, such as tests of normality and adjustment for multiple comparisons
☐	☒	A full description of the statistical parameters including central tendency (e.g. means) or other basic estimates (e.g. regression coefficient) AND variation (e.g. standard deviation) or associated estimates of uncertainty (e.g. confidence intervals)
☐	☒	For null hypothesis testing, the test statistic (e.g. *F*, *t*, *r*) with confidence intervals, effect sizes, degrees of freedom and *P* value noted<br>*Give P values as exact values whenever suitable.*
☐	☒	For Bayesian analysis, information on the choice of priors and Markov chain Monte Carlo settings
☐	☒	For hierarchical and complex designs, identification of the appropriate level for tests and full reporting of outcomes
☐	☒	Estimates of effect sizes (e.g. Cohen's *d*, Pearson's *r*), indicating how they were calculated

Our web collection on statistics for biologists contains articles on many of the points above.

Software and code

Policy information about availability of computer code

Data collection	The MGnify API (https://www.ebi.ac.uk/metagenomics/api/latest/) assessed via Python 3 using the jsonapi_client library (https://github.com/qvantel/jsonapi-client).
Data analysis	CheckM v1.0.7; iTOL; Python 3 using the following libraries: Numpy, Scipy, matplotlib, pandas, seaborn, scikit-learn v0.21.3, EMDUnifrac, scikit-bio v0.5.5, GSEApy v0.7.3, ete3 v3.1.1, Cartopy v0.17.0.<br>All custom code used for this manuscript is available on Zenodo at DOI: 10.5281/zenodo.7651594. A stand-alone script to calculate SNB is available on https://github.com/MGXlab/social_niche_breadth.

For manuscripts utilizing custom algorithms or software that are central to the research but not yet described in published literature, software must be made available to editors and reviewers. We strongly encourage code deposition in a community repository (e.g. GitHub). See the Nature Portfolio guidelines for submitting code & software for further information.

Data

Policy information about availability of data

All manuscripts must include a data availability statement. This statement should provide the following information, where applicable:

- Accession codes, unique identifiers, or web links for publicly available datasets
- A description of any restrictions on data availability
- For clinical datasets or third party data, please ensure that the statement adheres to our policy

All data analysed during this study are included in this article and its Supplementary Data Files, or available in public repositories. The selected samples from the MGnify resource (https://www.ebi.ac.uk/metagenomics/) are described in Supp. Data File 1. The cleaned taxonomic profiles based on this data are available in Supp. Data File 2. The selected genomes from the PATRIC database (https://www.bv-brc.org/) are described in Supp. Data file 5. Measures derived from the PATRIC genomes, and the EGGO (https://github.com/jlw-ecoevo/eggo) and TimeTree (https://timetree.org/) database are available in Supp. Data File 3.

Human research participants

Policy information about studies involving human research participants and Sex and Gender in Research.

Reporting on sex and gender	*Use the terms sex (biological attribute) and gender (shaped by social and cultural circumstances) carefully in order to avoid confusing both terms. Indicate if findings apply to only one sex or gender; describe whether sex and gender were considered in study design whether sex and/or gender was determined based on self-reporting or assigned and methods used. Provide in the source data disaggregated sex and gender data where this information has been collected, and consent has been obtained for sharing of individual-level data; provide overall numbers in this Reporting Summary. Please state if this information has not been collected. Report sex- and gender-based analyses where performed, justify reasons for lack of sex- and gender-based analysis.*
Population characteristics	*Describe the covariate-relevant population characteristics of the human research participants (e.g. age, genotypic information, past and current diagnosis and treatment categories). If you filled out the behavioural & social sciences study design questions and have nothing to add here, write "See above."*
Recruitment	*Describe how participants were recruited. Outline any potential self-selection bias or other biases that may be present and how these are likely to impact results.*
Ethics oversight	*Identify the organization(s) that approved the study protocol.*

Note that full information on the approval of the study protocol must also be provided in the manuscript.

Field-specific reporting

Please select the one below that is the best fit for your research. If you are not sure, read the appropriate sections before making your selection.

☐ Life sciences ☐ Behavioural & social sciences ☒ Ecological, evolutionary & environmental sciences

For a reference copy of the document with all sections, see nature.com/documents/nr-reporting-summary-flat.pdf

Ecological, evolutionary & environmental sciences study design

All studies must disclose on these points even when the disclosure is negative.

Study description	Generalists can survive in many environments whereas specialists are restricted to a single environment. Although a classical concept in ecology, niche breadth has remained challenging to quantify for microbes because it depends on an objective definition of the environmental conditions. Here, by defining the environment of a microbe as the community it resides in, we integrated information from over 22 thousand environmental sequencing samples to derive a quantitative measure of the niche, which we call 'social niche breadth'. At the level of genera, we explored niche range strategies throughout the prokaryotic tree of life. We found that social generalists include opportunists that stochastically dominate local communities, while social specialists are stable but low in abundance. Social generalists have a more diverse and open pan genome than social specialists, but we found no global correlation between social niche breadth and genome size. Instead, we observed two distinct evolutionary strategies, where specialists have relatively small genomes in habitats with low local diversity, but relatively large genomes in habitats with high local diversity. Together, our analysis shines data-driven light on microbial niche range strategies.
Research sample	22,518 selected from the MGnify database.
Sampling strategy	A maximum of 1,000 random samples per biome annotated with pipeline version 4.1 of MGnify.
Data collection	MGnify API.

Timing and spatial scale	2019-08-20
Data exclusions	None
Reproducibility	We only used publicly available data.
Randomization	Not applicable, because we analysed the full set of environmental sequencing datasets together.
Blinding	Not applicable, because our data-driven approach was specifically designed to avoid interpreting data according to categorical annotations, which we show in the paper are both arbitrary and redundant.

Did the study involve field work? ☐ Yes ☒ No

Reporting for specific materials, systems and methods

We require information from authors about some types of materials, experimental systems and methods used in many studies. Here, indicate whether each material, system or method listed is relevant to your study. If you are not sure if a list item applies to your research, read the appropriate section before selecting a response.

Materials & experimental systems

n/a	Involved in the study
☒	☐ Antibodies
☒	☐ Eukaryotic cell lines
☒	☐ Palaeontology and archaeology
☒	☐ Animals and other organisms
☒	☐ Clinical data
☒	☐ Dual use research of concern

Methods

n/a	Involved in the study
☒	☐ ChIP-seq
☒	☐ Flow cytometry
☒	☐ MRI-based neuroimaging
